# Histaminergic regulation of seasonal metabolic rhythms in Siberian hamsters

**DOI:** 10.1016/j.physbeh.2011.02.035

**Published:** 2011-06-01

**Authors:** Helen I'Anson, Preeti H. Jethwa, Amy Warner, Francis J.P. Ebling

**Affiliations:** a Biology Department, Washington and Lee University, Lexington, VA, USA; b School of Biomedical Sciences, University of Nottingham, UK; c Division of Nutritional Sciences, School of Biosciences, University of Nottingham, UK

**Keywords:** Histamine, Hypothalamus, Body weight, Food intake, Thioperamide, Melanin concentrating hormone

## Abstract

We investigated whether histaminergic tone contributes to the seasonal catabolic state in Siberian hamsters by determining the effect of ablation of histaminergic neurons on food intake, metabolic rate and body weight. A ribosomal toxin (saporin) conjugated to orexin-B was infused into the ventral tuberomammillary region of the hypothalamus, since most histaminergic neurons express orexin receptors. This caused not only 75–80% loss of histaminergic neurons in the posterior hypothalamus, but also some loss of other orexin-receptor expressing cells e.g. MCH neurons. In the long-day anabolic state, lesions produced a transient post-surgical decrease in body weight, but the hamsters recovered and maintained constant body weight, whereas weight gradually increased in sham-lesioned hamsters. VO_2_ in the dark phase was significantly higher in the lesioned hamsters compared to shams, and locomotor activity also tended to be higher. In a second study in short days, sham-treated hamsters showed the expected seasonal decrease in body weight, but weight remained constant in the lesioned hamsters, as in the long-day study. Lesioned hamsters consumed more during the early dark phase and less during the light phase due to an increase in the frequency of meals during the dark and decreased meal size during the light, and their cumulative food intake in their home cages was greater than in the control hamsters. In summary, ablation of orexin-responsive cells in the posterior hypothalamus blocks the short-day induced decline in body weight by preventing seasonal hypophagia, evidence consistent with the hypothesis that central histaminergic mechanisms contribute to long-term regulation of body weight.

## Graphical abstract

**Figure:**
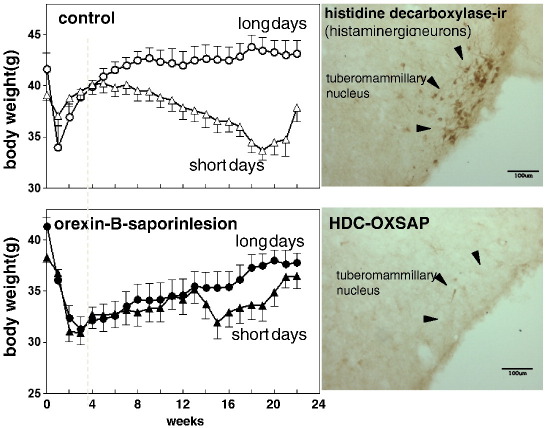

**Research highlights:**

► Saporin-orexin-B toxin ablated ~ 80% of tuberomammillary histaminergic neurons. ► Lesions disrupted seasonal rhythms of appetite and energy metabolism. ► Central histaminergic mechanisms contribute to long-term regulation of body weight.

## 1 Introduction

Siberian hamsters (*Phodopus sungorus*) have become widely used as a rodent model to understand long-term control of body weight because they undergo major changes in physiology and behavior enabling them to survive annual cycles of climatic and environmental change [1,2]. Siberian hamsters anticipate seasonally reduced food availability during winter by a short photoperiod-induced decrease in voluntary appetite, resulting in the loss of up to 40% of body mass, mostly in the form of intra-abdominal fat reserves [3]. We seek to understand the hypothalamic mechanisms underlying this natural hypophagia and weight loss, as they may provide amenable targets for therapeutic regulation of body weight. Recent investigations of differential hypothalamic gene expression in hamsters exposed to changing photoperiods have revealed down-regulation of the gene encoding the H3 histaminergic receptor in the dorsomedial posterior arcuate nucleus (dmpARC) in the short day state [4]. As this receptor is generally considered to be a presynaptic autoreceptor and/or heteroreceptor this would be consistent with increased histaminergic activity. Indeed, a recent electrophysiological study has demonstrated increased neuronal activity in the dmpARC in hamsters in short days which is dependent on down-regulation of the H3 receptor [5]. The overall aim of this study therefore was to test the hypothesis that increased histaminergic activity contributes to the development of the short-day catabolic state.

Many previous lines of evidence have identified a role for histamine in the control of energy partitioning via the regulation of feeding, locomotion, thermoregulation and hibernation [6,7]. Previous research has also shown that histamine is involved in the circadian rhythm of locomotor activity and exploratory behavior [8]. Histamine regulates metabolic homeostatic mechanisms including food and water consumption by acting on receptors within the hypothalamus. Blockade of post synaptic H_1_ receptors induces weight gain, and mice lacking the H_1_ receptor exhibit a phenotype of inadequate leptin modulation of appetite, as well as increased food consumption [9]. Blockade of histamine synthesis also increases weight gain, and obese Zucker rats exhibit low concentrations of hypothalamic histamine [10]. It is therefore unsurprising that there has been considerable interest in the development of agents which enhance histaminergic neurotransmission as anti-obesity treatments [11–13].

We have previously used a pharmacological approach to investigate the role of histaminergic mechanisms in the short-term control of food intake and energy metabolism. These studies have confirmed that acute manipulation of histaminergic neurotransmission affects food intake in the hamster [14], but the pharmacological approaches used in that study are of limited value for chronic studies. We have therefore used the technique of neurotoxic lesion of histaminergic neurons within the posterior hypothalamus to manipulate histaminergic activity in the hamster. The majority of histaminergic neurons possess orexin B receptors [15] which were targeted using intracranial microinjection of a saporin-orexin B receptor conjugate. This conjugate binds to OX1R and preferentially to OX2R orexin receptors on the cell surface and is internalized. Upon internalization, the conjugate dissociates and the saporin is translocated back to the neuron cell body where it destroys ribosomes and hence the entire cell, including all neuronal processes to other brain regions [16,17]. Although it is clear that some other neuronal phenotypes in the lateral and posterior hypothalamus also express orexin receptors, including orexin producing neurons themselves and melanin concentrating hormone (MCH) neurons [18,19], we predicted that by directing the neurotoxin into the ventral tuberomammillary region of the posterior hypothalamus we would preferentially ablate histaminergic neurons, allowing us to determine the role of such neurons in the regulation of seasonal rhythms of energy intake and expenditure.

## 2 Materials and methods

### 2.1 Animals

Adult male hamsters were obtained from a colony of Siberian hamsters (*Phodopus sungorus*) maintained at the University of Nottingham [20]. They were housed in individual cages under controlled temperature (21 ± 1 °C) and lighting conditions (16 h light/8 h dark cycle; lights off at 11:00 h) with *ad libitum* access to food (Harlan Teklad diet 2019) and water. After surgery and for the remainder of the study, pelleted food was placed inside the cage as well as in the feeding hoppers. In addition, ground food was placed inside the home cage for the week prior to testing in metabolic cages. We used this strategy to assist recovery from the initial surgery and to habituate the hamsters to the ground diet in preparation for collection of metabolic data in each study where only ground diet would be available. All animal procedures were approved by the University of Nottingham Local Ethical Review Committee and were carried out in accordance with the Animals Scientific Procedures Act (UK) 1986.

### 2.2 Surgical procedures

Hamsters were randomly assigned to two groups so that mean body weight was not different between groups. They were anesthetized with a mixture of ketamine (Vetalar 100 mg/kg ip, Forte Dodge Animal Health, Southampton, UK) and medetomidine (Dormitor 250 μg/kg ip, Pfizer, Kent, UK). Analgesia was maintained via subcutaneous (sc) injection of carprofen (20 mg/kg Rimadyl, Pfizer, Kent, UK) and fluid replacement (0.9% saline) before surgery. Infusions of the 28 amino acid orexin-B peptide (also known as hypocretin-2) conjugated to saporin (OXSAP, Chemicon International, Inc., Temecula, CA, 92 ng/μl in phosphate buffer, pH 7.4) or control solution containing an equivalent amount of unconjugated saporin (SAP, Advanced Targeting Systems, 19.2 ng/μl in phosphate buffer, pH 7.4) were made bilaterally in the tuberomammillary posterior hypothalamic region at the coordinates AP: − 0.4 mm from the bregma, ML: ± 0.5 mm, and DV: 6.0 mm below the dura, using a drawn glass capillary micropipette (20–30 μm tip diameter) connected to a nanoliter injector delivering 200 nl per infusion (World Precision Instruments, LTD, Herts, UK). Following surgery, the animals were given the anesthetic reversal atipamezole (Antisedan 1 mg/kg ip, Pfizer, Kent, UK). Daily augmentation of fluids (0.9% physiological saline containing Rimadyl analgesia) was continued during a recovery period of seven days.

### 2.3 Determination of metabolic parameters

Study 1 **Effect of OXSAP lesions on food intake, body weight and metabolic parameters in hamsters in long photoperiods**

Hamsters treated with OXSAP (n = 13) or SAP (n = 7) remained individually housed in long photoperiods for the duration of the study. Food intake in the home cage and body weight were monitored weekly, and 17 weeks after the study was initiated metabolic parameters (oxygen consumption, carbon dioxide production, and respiratory exchange ratio), locomotor activity and various parameters of eating behavior (frequency and duration of feeding bouts, and food consumption per bout and total food intake) were measured using a Columbus Instruments Comprehensive Lab Animal Monitoring System (CLAMS: Linton Instrumentation, Linton, UK/Columbus Instruments, Columbus, OH) as previously described [21]. This is a modified open circuit calorimeter, and the configuration used consisted of 8 chambers in which hamsters were studied individually. In order to monitor all the hamsters, three consecutive 48 h recording sessions were carried out, with hamsters from both groups divided between all three sessions. Centrally positioned feeders containing powdered hamster chow allowed meal size and the timing and duration of each feeding bout to be recorded. For subsequent analysis a “meal” was defined as a bout of food intake of greater than 0.02 g. The system was operated with an air intake of 0.6 L per minute per chamber, and an extracted outflow of 0.4 L per minute. The chamber volume of ~ 2.7 L allows approximately 14 air changes per hour. A sample of air was extracted from each chamber every 9 min for sequential analysis of CO_2_ then O_2_. The room air (i.e., input air to the chambers) was similarly analyzed every 9 min. Water was provided by dropper bottles, but intake was not recorded. Activity was recorded when two or more consecutive infra red beams positioned approximately 2 cm apart were broken. All measurements were taken at an ambient temperature of 21–22 °C.

Three weeks after this initial CLAMS session while the hamsters were still exposed to LD, metabolic rate, locomotor activity and feeding behaviors in all 7 SAP hamsters and in 9 randomly selected OXSAP hamsters were monitored for two further sessions in which they were treated with either vehicle or an H3R antagonist (thioperamide, 30 mg/kg, ip) as a probe to determine if a significant decrease in histaminergic tone had occurred. Each hamster received thioperamide or saline (0.9%) at the start of the dark phase in a cross-over design after having been habituated in the CLAMS apparatus for at least 24 h.

Study 2 **Effect of OXSAP lesions on food intake, body weight and metabolic parameters in hamsters in short photoperiods**

Following a 3-week recovery from surgery, hamsters treated with OXSAP (n = 14) or SAP (n = 8) were moved to a short day (SD) photoperiod (8 h light/16 h dark cycle; lights off at 11:00 h). Food intake, body weight and pelage score were monitored weekly. Pelage was scored where 4 = full summer coat and 1 = complete winter coat [22]. At 9 and 17 weeks after transfer to short days, all hamsters were placed into the CLAMS to monitor metabolic and behavioral parameters as described above.

#### 2.3.1 Immunohistochemistry and quantification of HDC and MCH-immunoreactive cell bodies

The hamsters in Study 1 were euthanized by injection of pentobarbital sodium (Euthatal; Rhone Merieux, Harlow, UK) and transcardially perfused with 0.01 M phosphate buffered saline (PBS, pH 7.2) followed by 4% paraformaldehyde in 0.1 M phosphate buffer (pH 7.2). Brains were then cryoprotected overnight in 20% sucrose and coronal 40 μm sections were cut on a freezing microtome. Intrascapular brown adipose tissue fat pads, testes, epididymides and epididymal fat pads were removed and weighed. Measurement of carcass composition in other studies has revealed that epididymal fat pad weight is directly proportional to whole carcass fat content (Murphy and Ebling, unpublished data). Every 4th section was processed for either Nissl stain (cresyl violet) or for immunohistochemical detection of histidine decarboxylase (HDC). This is the rate limiting step in histamine biosynthesis and therefore serves as marker for histaminergic neurons [23]. A third series was immunostained for melanin concentrating hormone (MCH). These peptidergic neurons are located in the lateral hypothalamus. Loss of MCH-ir neurons and orexin-ir neurons has been reported after infusion of the orexin B-saporin conjugate into the lateral hypothalamus of the rat [18,24], so changes in the abundance and distribution of MCH-ir neurons should provide a marker for the extent of the OXSAP lesion which was aimed at the tuberomammillary complex. Brain tissue from the hamsters in Study 2 could not be analyzed because of a mechanical failure of the freezer in which they were stored.

**Fig. 1:**
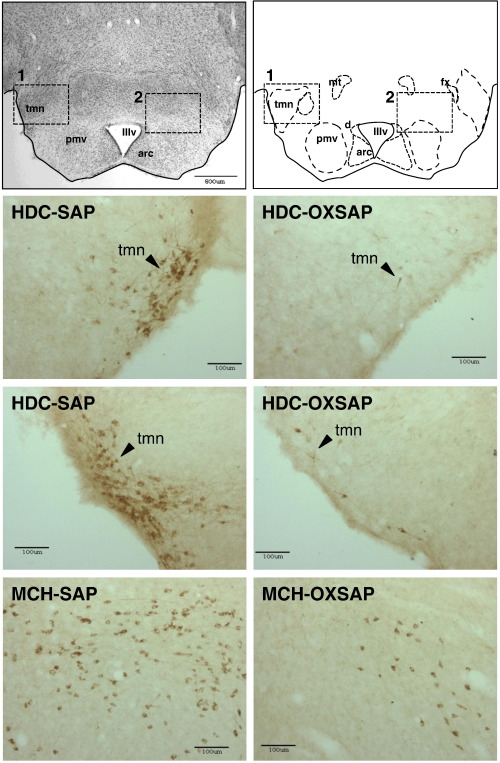
Representative examples of immunostaining for histidine decarboxylase (HDC) and melanin concentrating hormone (MCH) in coronal sections through the posterior hypothalamus from hamsters receiving control infusions of saporin (SAP, left) or infusions of a saporin-orexin B conjugate (OXSAP: right) in Study 1. Scale bars = 100 μm. Upper panels (scale bar = 500 μm) indicate the regions depicted for the HDC immunostaining (region 1) and the MCH immunostaining (region 2), but the actual sections for the MCH staining were taken 100–200 μm rostral to the coronal section depicted. IIIv: mammillary recess of the third ventricle, mt: mammillothalamic tract, fx: fornix, tmn: tuberomammillary nucleus, arc: posterior arcuate nucleus, d: dorsomedialposterior arcuate nucleus, and pmv: premammillary nucleus. Note the almost complete loss of HDC-ir perikarya following OXSAP infusion, but only the partial loss of MCH-ir soma.

Immunohistochemical staining was carried out using standard avidin–biotin-peroxidase techniques. Briefly, sections were treated with 0.5% hydrogen peroxide for 10 min to remove endogenous peroxidase activity, then washed (2 × 5 min) in 0.01 M PBS and incubated for 1 h in 5% normal goat serum made in 0.01 M PBS, 1% bovine serum albumin (BSA) and 0.3% Triton-X. The blocking solution was removed from the tissue and the sections were incubated for 24 h at 4 ºC in rabbit polyclonal anti-HDC (IDS Immunodiagnostics, Boldon, UK) at a working concentration of 1:40,000 or rabbit polyclonal anti-MCH ([25], 1:1000) made in 2% normal goat serum-PBS with 1% BSA and 0.3% Triton-X. The primary antiserum was removed and the sections were washed and incubated for 1 h at room temperature in biotinylated goat anti-rabbit IgG (1:600 in 0.01 M PBS with 0.3% BSA and 0.1% Triton-X, Jackson ImmunoResearch Laboratories). The tissue was subsequently washed (2 × 5 min), incubated with avidin–biotin horseradish peroxidase (“ABC” from Vectastain Elite Kit) for 1 h at room temperature, washed again (3 × 5 min) and reacted for visualization of HDC-ir using nickel-intensified diaminobenzidine in the peroxidase reaction to produce a dark reaction product. Sections were then mounted on slides and cover-slipped for microscopic evaluation. All antisera used in the experiment were titrated prior to use to determine optimal concentrations. Standard controls for specificity of primary antibodies were used, including the incubation of the tissue with normal instead of immune serum and pre-incubation of the immune serum with the antigen prior to its application to tissue. Histological sections presented in Fig. 1 were viewed using a Leica DMRB microscope and captured using Openlab software (Improvision, Coventry, UK). Brightness and contrast only were altered digitally in some cases using Adobe Photoshop v5.5.

**Fig. 2:**
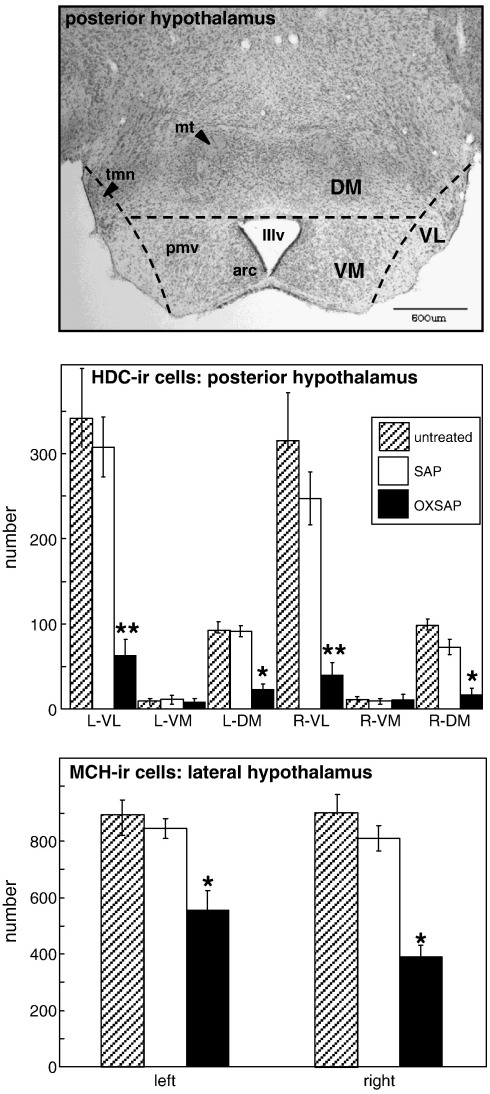
Histological analysis of the effects of the saporin-orexin B conjugate in Study 1. Top: a coronal section stained with cresyl violet through the posterior hypothalamus of a hamster indicating how the subdivisions for counting immunoreactive cells were designated, scale bar = 500 μm. Middle: numbers of histidine decarboxylase (HDC) immunoreactive cells in the ventrolateral (VL), ventromedial (VM) and dorsomedial (DM) divisions of the posterior hypothalamus on the left (L) and right (R) sides of the brain in untreated hamsters (hatched bars), hamsters receiving control infusions of saporin (open bars) or infusion of a saporin-orexin B conjugate (solid bars). Bottom: numbers of melanin concentrating hormone (MCH) immunoreactive cells in the lateral hypothalamus (LH) on the left and right sides of the brain. Values are group mean ± SEM, n = 4 untreated, n = 7 SAP and n = 9 OXSAP. **P < 0.0001 and *P < 0.001 vs SAP-treated group. Abbreviations as per the legend for Fig. 1.

HDC-ir cell bodies were quantified in every 4th section through the hypothalamus rostrally from the anterior arcuate nucleus to the mammillary bodies. Discrete subpopulations of histaminergic neurons (E1–E5) have been described in the tuberomammillary region of the posterior hypothalamus of the rat brain [23,26], but these subdivisions were not obvious in the hamster. Therefore, the hypothalamus was subdivided into ventro-lateral, ventro-medial and dorso-medial regions (Fig. 2), and HDC-ir magnocellular neuron cell bodies were counted bilaterally in these regions. The distribution of MCH-containing cell bodies has been reported to be restricted to the lateral hypothalamus in the rat [27], and we observed a very similar distribution in Siberian hamsters. Therefore, MCH-ir was quantified in every 4th section and cell bodies were counted bilaterally from the anterior arcuate nucleus to the rostral border of the tuberomammillary nuclear region of the hypothalamus.

#### 2.3.2 Data analysis

Statistical analyses were carried out using Prism v4.0 (GraphPad Software, San Diego, CA). For analysis of data obtained from the CLAMS apparatus, mean hourly values for VO_2_, VCO_2_, respiratory exchange ratio (RER), locomotor activity, and parameters of food intake were calculated for the second 24 h that the animals were housed in the CLAMS system. Since thioperamide has a relatively short duration of action in Siberian hamsters [14], the analyses compared the experimental variables in the two hour period following vehicle or thioperamide treatment. Data were analyzed using a two factor analysis of variance (ANOVA) with repeated measures with Bonferroni tests for post-hoc comparisons (Prism). Comparisons resulting in a P < 0.05 were considered significant. Data are presented as mean ± standard error of the mean.

## 3 Results

### 3.1 Effect of OXSAP treatment on abundance of HDC-ir and MCH-ir cells in the hypothalamus

A comprehensive immunohistochemical analysis was carried out on 9 randomly selected brains from OXSAP-treated hamsters and from 7 brains from SAP-treated controls, and these were compared to sections from an age-matched group of 4 untreated male hamsters that were processed at the same time. In the SAP-treated and untreated groups, HDC-ir magnocellular neurons were located in the tuberomammillary region of the posterior hypothalamus (Fig. 1), but were not observed to be discretely localized into the five subgroups (E1–E5) that have previously been documented in the rat [23,26]. SAP-treatment did not affect the number of HDC-ir cell soma (Fig. 2 middle), however the numbers of HDC-ir magnocellular neurons were significantly reduced by 5-fold in the ventrolateral region (P < 0.0001) and by 4-fold in the dorsomedial regions (P < 0.002) of the posterior hypothalamus in OXSAP-treated hamsters (Figs. 1 and 2). Overall, fewer HDC-ir magnocellular neurons were observed in the ventromedial (VM) region of the posterior hypothalamus, and no significant effect of the OXSAP treatment was observed in this region. MCH-ir cell somata were widely distributed in the lateral hypothalamus (Fig. 1). The numbers of MCH-ir neurons were significantly reduced (P < 0.001) by 1.5- to 2-fold in the lateral hypothalamus of lesioned hamsters compared with sham hamsters (Figs.1 and 2).

Study 1 **Effect of OXSAP lesions on food intake, body weight and metabolic parameters in hamsters in long photoperiods**

**Fig. 3:**
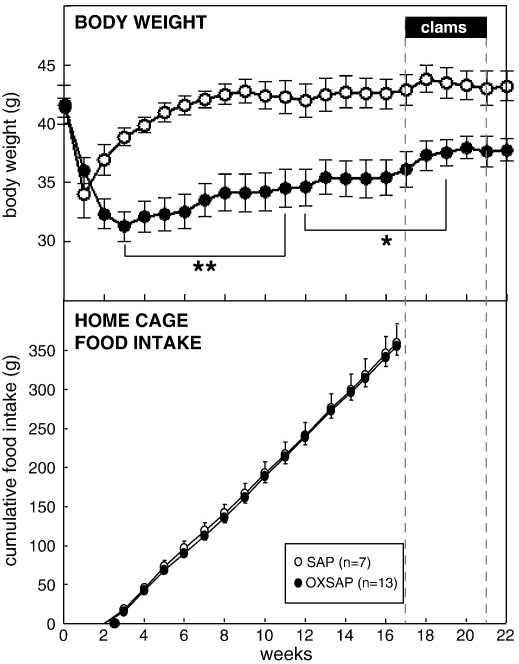
Study 1: body weight (upper panel) and cumulative food intake in the home cage (lower panel) of adult male hamsters which were maintained in long days and received bilateral infusions of saporin (SAP, open symbols) or a saporin-orexin B conjugate (OXSAP: closed symbols). Solid bars (clams) and dotted lines indicate the period when hamsters were removed from their home cages and studied in metabolic cages on three separate occasions (see main text), food intake was not monitored in their home cages during this time. Values are group mean ± SEM, n = 7 SAP and n = 13 OXSAP, *P < 0.05 and **P < 0.01 vs SAP group at the respective time point.

Hamsters in both groups lost weight following surgery, however, there were generally no signs of stress or trauma in the animals during this period and recovery was rapid in both groups in terms of eating, grooming and activity levels. After one week of weight loss, the control hamsters infused with SAP began to gain weight, and continued on a positive growth trajectory (Fig. 3, top). In contrast, hamsters infused with OXSAP continued to lose weight for up to 3 weeks post-surgery, thereafter they gradually regained weight (Fig. 3, top), but were significantly lighter than the SAP-treated controls throughout the study (Fig. 3, top). Maintenance of a lower body weight in the OXSAP-treated hamsters occurred despite their actual food intake in the home cage being similar to that of the SAP-treated hamsters (Fig. 3, bottom).

**Fig. 4:**
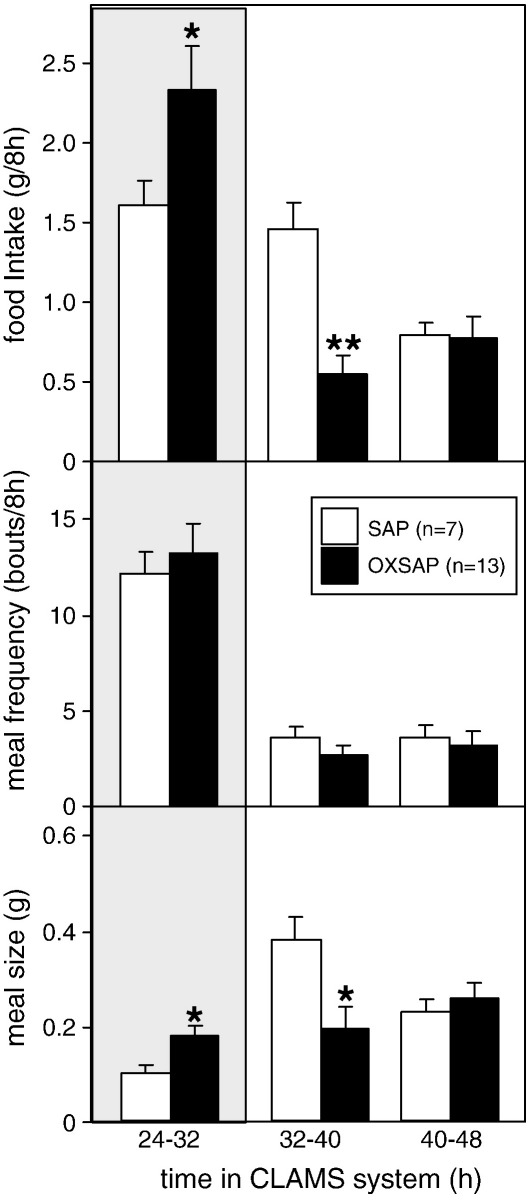
Study 1: food intake (upper panel), meal frequency (middle panel) and meal size (lower panel) of adult male hamsters in the CLAMS apparatus maintained in long days (16L:8D) for 17 weeks after receiving bilateral infusions of saporin (SAP, open bars) or a saporin-orexin B conjugate (OXSAP: closed bars). Values are group mean ± SEM for 8 h epochs, n = 7 SAP and n = 13 OXSAP. Shaded background indicates the dark phase. *P < 0.05 and **P < 0.01 vs SAP group.

**Fig. 5:**
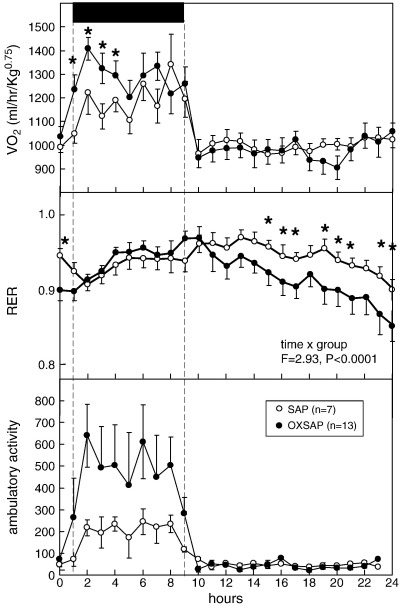
Study 1: oxygen uptake (VO_2_: upper panel), respiratory exchange ratio (RER: middle) and locomotor activity (lower panel) of adult male hamsters in the CLAMS apparatus maintained in long days after receiving bilateral infusions of saporin (SAP, open symbols) or a saporin-orexin B conjugate (OXSAP: closed symbols). Values are group mean ± SEM for 1 h epochs, n = 7 SAP and n = 13 OXSAP. Solid bar and broken lines indicate the dark phase. *P < 0.05 vs SAP group.

Patterns of food intake were also analyzed in the CLAMS system at 17 weeks post surgery (Fig. 4). Two factor ANOVA revealed a clear effect of time (F = 23.7, P < 0.0001), thus food intake was higher in both groups during the dark phase than during the light phase (Fig. 4, top) reflecting a significantly increased frequency of feeding (F = 70.5, P < 0.0001; Fig. 4, bottom). There was also a significant time × group interaction (F = 5.62, P = 0.0001), thus food intake in the OXSAP group was higher in the dark phase but lower in the first 8 h of the light phase (Fig. 4, top). This reflected significant differences in meal size (Fig. 4, bottom), as there were no significant differences in frequency of feeding between the groups (Fig. 4, middle). Metabolic rate as inferred from the VO_2_ measurements was significantly higher during the first part of the dark phase in the OXSAP-treated hamsters compared with SAP-treated controls (Fig. 5, top). The daily profile of fuel metabolism, as measured by respiratory exchange ratio (RER), also differed between the groups (Fig. 5, middle). Two factor ANOVA revealed not only a clear effect of time (F = 9.15, P < 0.0001) but also a significant group × time interaction (F = 2.93, P < 0.0001), thus RER values were similar during the dark phase, but lower during the light phase in the OXSAP-treated hamsters (Fig. 5, middle). Locomotor activity was much more variable between individuals; there was a trend (p = 0.09) towards higher activity during the dark phase in OXSAP-treated hamsters compared with the SAP-treated controls (Fig. 5 bottom), but no differences in activity levels were detected in the light phase (Fig. 5, bottom).

**Fig. 6:**
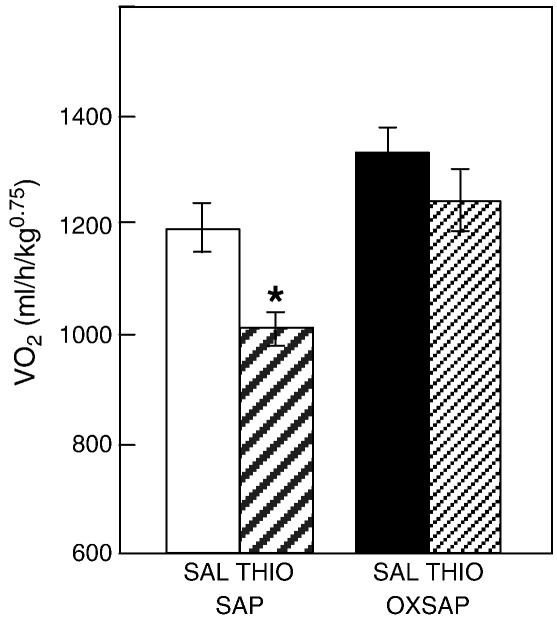
Study 1: VO_2_ in hamsters which had previously received bilateral infusions of saporin (SAP) or a saporin-orexin B conjugate (OXSAP) following saline vehicle treatment (SAL) or 30 mg/kg body weight thioperamide i.p. (THIO). Values are group mean ± SEM for the 2 h epoch following treatment, n = 7 SAP and n = 9 OXSAP. *P < 0.05 vs vehicle treatment.

Intraperitoneal injection of thioperamide (30 mg/kg) produced a significant (P < 0.05) decrease in VO_2_ for a two hour period in SAP-treated hamsters but not in the hamsters that had been lesioned with OXSAP (Fig. 6). No parameters of food intake nor locomotor activity were significantly altered by thioperamide treatment in either group (data not shown). At necropsy 22 weeks after the initial OXSAP/SAP infusion, there were no significant differences between the groups in the wet weight of the intra-scapular brown adipose tissue, testes or epididymides (Table 1), however the epididymal fat pad weight was significantly lower in OXSAP-treated hamsters (Table 1), consistent with the lower overall body weight (Fig. 3, Table 1).

Study 2 **Effect of OXSAP lesions on food intake, body weight and metabolic parameters in hamsters in short photoperiods**

**Fig. 7:**
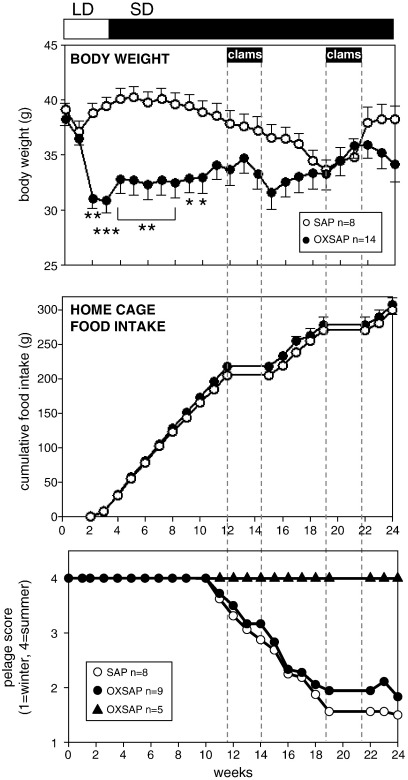
Study 2: body weight (top), cumulative food intake in the home cage (middle) and pelage score (bottom) of adult male hamsters transferred to short days and receiving bilateral infusions of saporin (SAP, open symbols) or a saporin-orexin B conjugate (OXSAP: closed symbols). Values are group mean ± SEM, group sizes as indicated, *P < 0.05, **P < 0.01, and ***P < 0.001 vs SAP group at the respective time point. Solid bars (clams) and dotted lines indicate periods when hamsters were removed from their home cages and studied in metabolic cages. Note that body weight decreased in the SAP-treated control hamsters for 16 weeks, but then began to spontaneously increase back to initial values, whereas body weight was more constant in the OXSAP group.

As would be expected, weight loss occurred in the SAP-treated control hamsters during short day exposure reaching a nadir at 16 weeks in short days (Fig. 7, top) followed by an increase representing a photorefractory response to day length, as has previously been documented many times in Siberian hamsters [28,29]. In contrast, although there was a post-surgical drop in body weight, the OXSAP-treated hamsters did not lose further weight with exposure to short day photoperiods, and slightly gained weight during the 20 week study period (Fig. 7, top). Their cumulative food intake in the home cage was slightly elevated over weeks 2–12 compared with SAP-treated hamsters (Fig. 7, middle: group × time interaction F = 2.29, P < 0.05). A full winter molt was observed in all SAP-treated hamsters (Fig. 7, bottom) and in 9 of 14 OXSAP hamsters (Fig. 7, bottom), though the remaining 5 hamsters in the OXSAP group remained in the summer pelage condition. As we typically find that 10–20% of hamsters in our colony fail to molt when exposed to short days, the number of hamsters showing a full molt was analyzed in the SAP vs OXSAP groups using a Chi-squared test. There was no statistically significant difference in the proportion of hamsters failing to molt (Chi^2^ = 3.70, P > 0.05). In addition, weekly body weight data were reanalyzed separately for the winter molt vs no molt subgroups of hamsters treated with OXSAP. The body weight profiles of these subgroups did not differ significantly from each other (main effect of subgroup F = 1.9, p = 0.19; subgroup × time interaction F = 0.84, p = 0.69), but both subgroups differed significantly from the SAP group (subgroup × time interactions: no molt subgroup F = 6.58, P < 0.0001; molt subgroup F = 6.24, P < 0.0001).

**Fig. 8:**
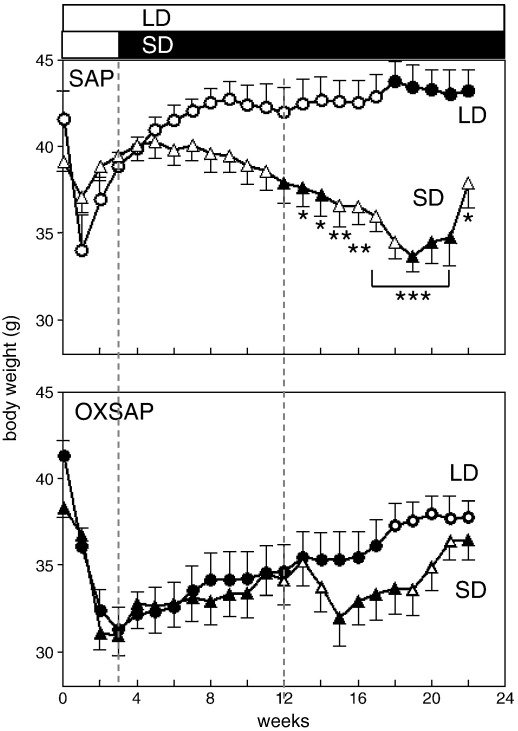
Top: a direct comparison of body weight in hamsters treated with SAP in LD (Study 1) vs SD (Study 2), and bottom: body weight in hamsters treated with OXSAP in LD (Study 1) vs SD (Study 2). Values are group mean ± SEM, re-plotted from Figs. 3 and 7. Body weights recorded while the hamsters were being tested in metabolic cages are denoted by closed symbols (SAP panel) and open symbols (OXSAP panel). *P < 0.05, **P < 0.01, and ***P < 0.001 vs SAP LD group.

When comparing body weight between Study 1 conducted in LD and Study 2 where the hamsters were transferred to SD, it is evident that there was a clear photoperiodic response in the SAP-treated hamsters after the initial post surgical recovery period (Fig. 8, top, photoperiod × week interaction F = 11.5, P < 0.0001). Body weight differed significantly between the two SAP groups by 10 weeks after the group in Study 2 had been switched to SD (Fig. 8, top). In contrast, body weight profiles were similar in the two groups of hamsters treated with OXSAP (Fig. 8, bottom); there was no significant photoperiod × week interaction suggesting that body weight was not being modified by photoperiod in these hamsters.

**Fig. 9:**
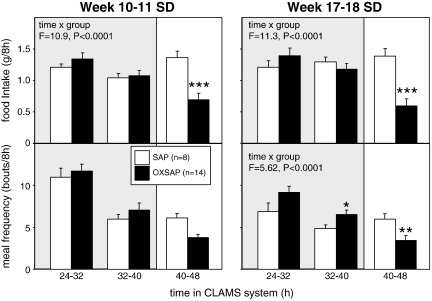
Study 2: food intake (upper panels) and meal frequency (lower panels) of adult male hamsters in the CLAMS apparatus after receiving bilateral infusions of saporin (SAP, open bars, n = 8) or a saporin-orexin B conjugate (OXSAP: closed bars, n = 14) after 10–11 weeks exposure to short days (left) and after 17–18 weeks exposure to short days (right). Values are group mean ± SEM for 8 h epochs, shaded background indicates the dark phase. *P < 0.05, **P < 0.01, and ***P < 0.001 vs SAP-treated group.

**Fig. 10:**
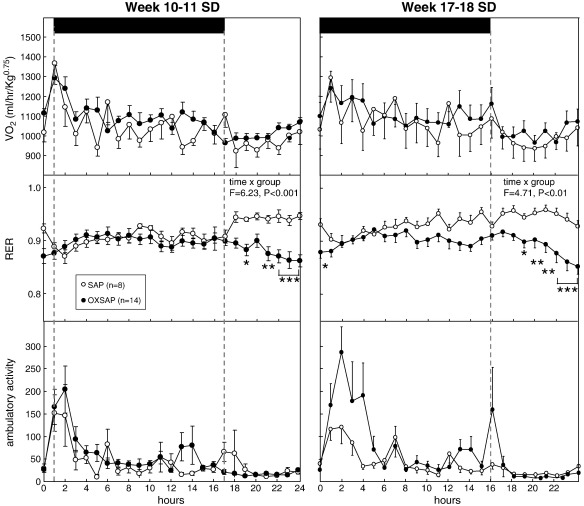
Study 2: oxygen uptake (VO_2_: upper panel), respiratory exchange ratio (RER: middle) and locomotor activity (lower panel) of adult male hamsters in the CLAMS apparatus maintained in short days after receiving bilateral infusions of saporin (SAP, open symbols, n = 8) or a saporin-orexin B receptor conjugate (OXSAP: closed symbol, n = 14 s) after 10–11 weeks exposure to short days (left) and after 17–18 weeks exposure to short days (right). Values are group mean ± SEM, n = 8 per group, *P < 0.05, **P < 0.01, and ***P < 0.001 vs SAP-treated group.

In general, daily variation in both ingestive (Fig. 9) and metabolic parameters (Fig. 10) was attenuated in amplitude in both groups of hamsters under the short day photoperiod, consistent with other observations we have made with the CLAMS [30]. The small increase in food intake in the OXSAP-treated hamsters measured in home cages was not observed when the hamsters were studied in the metabolic cages (Fig. 9). Two factor ANOVA revealed a significant interaction between group and time on both occasions (SD week 10–11, F = 10.9; week 17–18 F = 11.3, P < 0.001), suggesting that the OXSAP-treated hamsters had a decreased food intake in the light phase reflecting a decrease in the frequency of feeding bouts (Fig. 9, bottom). At both weeks 10–11 (F = 6.67, P < 0.0001) and weeks 17–18 (F = 5.86, P < 0.0001) in short days there was a significant effect of time on VO_2_ (Fig. 10 top), but no effect of group, though the large amplitude dark phase rise in metabolic rate that was observed in hamsters during long day photoperiods in the first study was not as apparent in hamsters in short days on either occasion (Fig. 10 top vs Fig. 5 top). Analysis of RER values revealed that on both occasions in short days there were significant group × time interactions (week 10–11: F = 6.23, P < 0.001, weeks 17–18 F = 4.71, P < 0.01), thus RER values were lower for parts of the daily cycle in the OXSAP-treated hamsters (Fig. 10). Finally, locomotor activity did not differ over a 24-hour period between lesioned and sham hamsters at 9 or 17 weeks in short days (Fig. 10, bottom). In addition, the marked elevation in night-time activity that was observed in hamsters during long day photoperiods (Fig. 5, bottom: note the 2-fold difference in scale vs Fig. 10 bottom) was reduced to a small elevation in activity at the onset of the dark phase in short day exposed hamsters (Fig. 10).

At necropsy 24 weeks after the initial OXSAP/SAP infusion and approximately 21 weeks after the hamsters were transferred to SD, epididymal fat pad weight in the OXSAP-treated hamsters was significantly reduced compared to that in the SAP controls (Table 1), consistent with their significantly lower body weight (Table 1). The wet weight of the intra-scapular brown adipose tissue, testes and epididymides did not differ significantly between the two groups (Table 1). The body weight and epididymal fat pad weight of the hamsters in the SAP control group were not dissimilar from those of the SAP-treated hamsters that had been housed in LD in Study 1, indicating that by 21 week in SD these hamsters had started to become refractory and were regaining body weight (see also Fig. 8 upper panel). The wet weight of the testes and epididymides was significantly lower than those in the SAP-treated hamsters in Study 1 indicating that recrudescence of the reproductive axis was not complete at this time.

## 4 Discussion

The major finding from the current studies is that loss of neurons in the lateral and posterior hypothalamus which express orexin receptors disrupts seasonal cycles of energy metabolism in Siberian hamsters. The impaired seasonal response is particularly striking when directly comparing the two studies (Fig. 8). In long-day summer photoperiods which promote an anabolic state, hamsters with such lesions showed an impaired anabolic response compared to sham-lesioned controls, body weight remaining significantly lower than in the control hamsters (Fig. 3). Correspondingly, in short-day winter photoperiods which promote a catabolic state, hamsters with such lesions showed an impaired catabolic response compared to sham-lesioned controls, body weight remaining relatively stable in the lesioned hamsters whereas the characteristic long-term cycle of weight loss and then weight gain was observed in the sham-treated hamsters (Fig. 7). We recognize that the capacity for the OXSAP-treated hamsters to show a catabolic response was somewhat attenuated because the immediate effects of the infusion surgery was a period of acute weight loss. However, we would have expected that further weight loss could occur because we have previously observed body weights of substantially less than 30 g in male Siberian hamsters [31,32], Even if no further body weight loss were possible in the OXSAP-SD group, one might still have expected body weight to have remained static at 30 g if they were responding to SD rather than gradually increasing as was observed. This increase contrasted with the profile for the SAP-treated hamsters in SD which showed a gradual decrease in body weight over the same time period. In fact the similarity in body weight profiles of the OXSAP-treated groups in SD and LD was striking (Fig. 8); regardless of photoperiod there was a gradual increase in body weight after the post-surgical decrease in OXSAP-treated hamsters, indicating a lack of response to the SD photoperiod.

The impaired long-day anabolic response in hamsters treated with OXSAP appears to reflect an increased energy expenditure. Oxygen consumption was significantly increased, at least for the first part of the dark phase, whereas food intake in the home cage and in the metabolic cages did not differ from the SAP-treated control hamsters in LD. This increase in oxygen consumption may reflect in part an increase in locomotor activity in the dark phase in the OXSAP-treated hamsters. There were also subtle differences in the temporal pattern of feeding as the OXSAP-treated hamsters consumed significantly more during the dark phase but significantly less during the early light phase. The increase in activity during the early dark phase may be a reflection of the increased food intake at this time. The lower food intake during the light phase was associated with a significantly lower RER, thus greater reliance on oxidation of fat depots probably underlies the reduced body weight in the OXSAP-treated hamsters as compared to the SAP-treated controls in LD.

In contrast, the impaired winter catabolic response in OXSAP-treated hamsters in Study 2 appears to relate to a slightly attenuated short-day induced suppression of food intake, as intake of pelleted food in the home cage became significantly greater in the OXSAP-treated hamsters at the time when the control group began to display the characteristic short-day induced weight loss. This difference in food intake in the home cage was not reflected by the observations in the metabolic cages, in fact the OXSAP-treated hamsters actually had a decreased food intake in the light phase (though not in the dark phase) reflecting a decrease in the frequency of feeding bouts. There were no significant differences in oxygen consumption on either of the sampling occasions, though again there was a lower RER in the light phase in the OXSAP-treated hamsters which may reflect their reduced food intake and greater reliance on fat metabolism at this phase. Given that body weight is a reflection of energy balance over many weeks, whereas the observations in the CLAMS apparatus reflect physiology over just 48 h in a novel environment, we infer that the principal effect of the OXSAP lesion was to protect against winter hypophagia.

One explanation for our observations that the OXSAP-induced lesions blocked both long-day induced anabolic responses and blocked short-day induced catabolic responses is that the lesions interfered with the actual transduction of photoperiodic information to the brain, that is, the hamsters were rendered ‘blind’ to the ambient photoperiod. Lesions of the suprachiasmatic nucleus or other components of the nocturnal melatonin rhythm generation-response pathway clearly have this effect in Syrian [33] and Siberian [34] hamsters. However we consider it unlikely that the current lesions affected transduction of photoperiodic information because we observed a normal molt to white winter pelage in the majority of the OXSAP-treated hamsters moved to short days (Fig. 7). Moreover, there were no differences in the food intake or body weight profiles of the OXSAP-treated hamsters which did molt normally and those which failed to molt. Colonies of Siberian hamsters normally contain individuals which fail to show the complete range of normal short day responses, so to have some individuals which retained agouti summer pelage is not unexpected [35,36].

While histaminergic neurons were our primary target for the OXSAP lesion, we also observed a significant decrease in the density of MCH-ir neurons in the lateral hypothalamus, particularly in more caudal regions. This suggests that diffusion of the toxin occurred away from its intended locus, as there are previous reports that the OXSAP toxin ablates MCH-producing neurons when infused into the lateral hypothalamus of rats [37]. On the basis of previous studies using this neurotoxin in the rat, we would also expect the partial loss of orexin-producing neurons [18,24,37] which are located in the same region as the MCH neurons in the lateral hypothalamus of the hamster [31]. The OXSAP toxin ablates both OX1R and OX2R-expressing cells, though it might be expected to act more potently on cells expressing the latter receptor as native orexin-B peptide shows a ten-fold higher affinity for OX2R compared to OX1R [38]. Both OX2R and OX1R are expressed in the posterior hypothalamus of the rat [39,40], but orexin and MCH-containing neurons appear to express OX1R [19] so may be less susceptible to the OXSAP than OX2R containing neurons.

Our previous observations of orexin gene expression have not provided any evidence that changes in this peptidergic pathway contribute to photoperiodic changes in appetite [31]. We have observed that intracerebroventricular administration of MCH does promote food intake in the Siberian hamster (Schuhler and Ebling, unpublished data), but have not observed seasonal changes in MCH-immunoreactive cells in the hypothalamus (Horan, Baker and Ebling, unpublished data). We consider it to be unlikely that the attenuation of both long-day induced anabolic responses and short-day induced catabolic responses could be explained solely by partial loss of MCH and/or orexin peptidergic pathways. Mice with selective loss of MCH neurons produced by specific neurotoxic lesions lose body weight due to hypophagia and hyperactivity [41], we did not observe hypophagia in OXSAP-lesioned hamsters maintained in long days, and found some evidence of relative hyperphagia in OXSAP-lesioned hamsters maintained in short days. In addition, rats in which an OXSAP lesion was aimed directly at the lateral hypothalamus exhibited narcoleptic-like sleep behavior [18]. While we did not measure core temperature in our animals, activity rhythms measured in the CLAMS did not indicate any disruption of activity patterns which might indicate inappropriate sleep, nor did we detect narcolepsy when we directly observed the hamsters during either the light or dark phase, and there was no evidence from the CLAMS for decreased locomotor activity during the dark phase in the OXSAP-treated hamsters in either long or short photoperiods.

A clear feature of our studies is that the effects of loss of orexin-responsive neurons are dependent upon the seasonal state of the hamsters. Although photoperiod-regulated changes in activity of MCH, orexin and other unknown phenotypes of neuron expressing OX1R/OX2R might explain this, for the reasons outlined above we favor the view that this is a reflection of changing endogenous histaminergic activity. Previous studies have demonstrated increased histamine synthesis and fiber density in the hypothalamus of hibernating ground squirrels in winter [7]. However increased histaminergic activity in the overwintering ground squirrel was associated with upregulation of the mRNA encoding H3R and H3 agonist binding in a variety of forebrain structures [42]. Likewise increased H3R gene expression has been observed in hypothalamic areas of Siberian hamsters during bouts of winter torpor as compared to non torpid hamsters [43]. Our own observations suggest down-regulation of H3R gene expression, but this response is highly localized to the dorsomedial posterior arcuate nucleus [4], and relates directly to increased neuronal activity in that structure [44]. Pharmacological studies indicate that H3R contributes to the regulation of food intake in hamsters [14]. As histamine cell bodies are located exclusively in the posterior hypothalamus in the rodent [26], the upregulation of H3R expression in other parts of the brain are more likely to relate to the suppression of activity of other aminergic systems than to reduced histamine release. We therefore conclude that the observation that short-day weight loss was attenuated in hamsters bearing OXSAP lesions in the posterior hypothalamus is consistent with the hypothesis that increased histaminergic activity contributes to the establishment of the winter catabolic state.

## Figures and Tables

**Table 1:** Tissue weights at the end of each study.

	Study 1 long days		Study 2 week 24 short days	
	SAP (control)	OXSAP	SAP (control)	OXSAP
n	7	13	8	13
Body weight (g)	41.8 ± 1.0	37.8 ± 1.0⁎	39.7 ± 1.1	31.0 ± 1.7⁎⁎
EPIFAT (mg)	735 ± 54	539 ± 61⁎	647 ± 55	402 ± 78⁎
Testes (mg)	379 ± 32	355 ± 30	293 ± 24⁎⁎⁎	253 ± 29
Epididymides (mg)	179 ± 20	160 ± 14	130 ± 10⁎⁎⁎	136 ± 0
BAT (mg)	149 ± 12	140 ± 26	136 ± 19	105 ± 19

EPIFAT: epididymal fat pad weight, BAT: intrascapular brown adipose tissue, pelage 4 = summer, and 1 = winter. Values are group mean ± SEM.

⁎ P < 0.05 vs SAP group in the respective study.

⁎⁎ P < 0.01 vs SAP group in the respective study.

⁎⁎⁎ P < 0.05 vs SAP group in Study 1.
